# Data-driven connectivity profiles relate to smoking cessation outcomes

**DOI:** 10.1038/s41386-024-01802-9

**Published:** 2024-01-27

**Authors:** Laura Murray, Blaise B. Frederick, Amy C. Janes

**Affiliations:** 1 Cognitive and Pharmacological Neuroimaging Unit, National Institute on Drug Abuse, Biomedical Research Center, 251 Bayview Blvd, Baltimore, MD 21224 USA; 2 McLean Imaging Center, McLean Hospital, 115 Mill Street, Belmont, MA 02478 USA; 3 Department of Psychiatry, Harvard Medical School, Boston, MA 02215 USA

**Keywords:** Addiction, Cognitive neuroscience

## Abstract

At a group level, nicotine dependence is linked to differences in resting-state functional connectivity (rs-FC) within and between three large-scale brain networks: the salience network (SN), default mode network (DMN), and frontoparietal network (FPN). Yet, individuals may display distinct patterns of rs-FC that impact treatment outcomes. This study used a data-driven approach, Group Iterative Multiple Model Estimation (GIMME), to characterize shared and person-specific rs-FC features linked with clinically-relevant treatment outcomes. 49 nicotine-dependent adults completed a resting-state fMRI scan prior to a two-week smoking cessation attempt. We used GIMME to identify group, subgroup, and individual-level networks of SN, DMN, and FPN connectivity. Regression models assessed whether within- and between-network connectivity of individual rs-FC models was associated with baseline cue-induced craving, and craving and use of regular cigarettes (i.e., “slips”) during cessation. As a group, participants displayed shared patterns of connectivity within all three networks, and connectivity between the SN-FPN and DMN-SN. However, there was substantial heterogeneity across individuals. Individuals with greater within-network SN connectivity experienced more slips during treatment, while individuals with greater DMN-FPN connectivity experienced fewer slips. Individuals with more anticorrelated DMN-SN connectivity reported lower craving during treatment, while SN-FPN connectivity was linked to higher craving. In conclusion, in nicotine-dependent adults, GIMME identified substantial heterogeneity within and between the large-scale brain networks. Individuals with greater SN connectivity may be at increased risk for relapse during treatment, while a greater positive DMN-FPN and negative DMN-SN connectivity may be protective for individuals during smoking cessation treatment.

## Introduction

There is substantial heterogeneity within psychopathology, such that no two individuals with the same diagnosis are identical. Indeed, the current diagnostic criteria for substance use disorders (SUD), including tobacco use disorder (TUD), specify that individuals must have a minimum of two out of eleven symptoms, meaning up to five individuals with TUD can share *no symptoms* in common beyond frequent nicotine use [1]. Yet, there may be some SUD symptoms and related biological processes (e.g., craving) that are common across many individuals with SUD [2], indicating that individuals with SUD may have both common and individual-specific processes that contribute to their SUD. Parsing psychiatric heterogeneity is essential for improving etiological models of TUD and improving treatment outcomes, as different treatment approaches may be needed for individuals who have distinct symptom profiles.

Heterogeneity also poses a problem for neuroscientists who seek to identify the neurobiological correlates of psychiatric disorders to improve treatment and prevention. Different clinical profiles may be linked to unique patterns of brain function and different responses to treatment. Yet, unless this heterogeneity is explicitly modeled, it remains unexplained variance that can hinder analyses. Indeed, standard neuroimaging analyses use nomothetic approaches to aggregate across subjects to identify patterns in brain function on average. However, these approaches may fail to describe any individual in the sample and may miss important biological mechanisms that are clinically important for one subgroup but not another [3]. However, idiographic approaches that exclusively examine intra-individual variation over time may not generalize to the population of interest. For clinical science to improve psychiatric outcomes for individual patients *and* at scale, it is critical to integrate nomothetic and idiographic approaches. Approaches that simultaneously model the general (i.e., present across the group), shared (i.e., present within subgroups), and person-specific patterns in imaging data can capture psychiatric heterogeneity more accurately while allowing for generalizable conclusions about psychiatric disorders such as TUD.

Researchers have become increasingly interested in understanding how disturbances in large-scale brain networks contribute to TUD. The triple-network model [4] proposes that dysfunction in the engagement/disengagement of the default mode network (DMN), salience network (SN), and frontoparietal network (FPN) contribute to psychopathology, including TUD [5]. The DMN includes the medial prefrontal cortex and posterior cingulate cortex and is involved in self-referential mentalization and autobiographical memory. The SN includes the anterior insula, dorsal anterior cingulate cortex, and nucleus accumbens and is involved in integrating internal and external information to guide behavior. The FPN includes the dorsolateral prefrontal cortex and posterior parietal cortex including the intraparietal sulcus and plays an important role in cognitive control and attention. Dysfunction in one core network can impact the other networks, highlighting the importance of both within- and between-network connectivity in psychopathology. Although differences in large-scale brain resting-state functional connectivity (rs-FC) have been identified across a variety of psychiatric disorders, including TUD [5, 6], it remains unclear to what extent person-specific connectivity patterns are related to important clinical outcomes (e.g., craving, difficulty maintaining abstinence), and whether neurobiologically-defined subgroups are linked to differences in these outcomes.

Group Iterative Multiple Model Estimation (GIMME) integrates nomothetic and idiographic approaches to parse heterogeneity within neuroimaging data. GIMME is a data-driven approach that uses unified structural equation models to create sparse connectivity maps to explain variation in resting-state data [7, 8]. The goals of GIMME are two-fold: (1) identify relationships between brain regions that are generalizable to the population of interest (i.e., group-level) and (2) identify reliable person-specific patterns of relations between brain regions that describe individuals. GIMME can also identify subgroups that share similar connectivity patterns, which is particularly helpful to parse heterogeneity within a diagnostic group, such as individuals with TUD.

Several studies have used GIMME to characterize person-specific and subgroup-level rs-FC features and link them to clinical variables. One study found that a GIMME-derived subgroup with high network heterogeneity and low network density (i.e., less connectivity between network nodes) had higher rates of childhood violence exposure, and that more severe childhood violence exposure was linked to lower within-network SN and between-network DMN-SN density [9]. Another study did not characterize subgroups, but found that individuals with a greater proportion of connections between the DMN and FPN (i.e., greater between-network density) had higher levels of psychopathic traits [10]. In a study of adults with depression, two GIMME-derived subgroups emerged, the largest subgroup displayed a pattern of DMN connectivity previously shown to be linked to depression. The other subgroup displayed an atypical connectivity pattern including no significant within-network DMN paths and a greater number of paths including the anterior cingulate. This smaller subgroup had more females and had higher rates of comorbid anxiety and recurrent depression [11]. This study highlights the importance of parsing heterogeneity within a diagnostic group, as the GIMME method identified a subgroup of individuals with a pattern of connectivity different from what traditional neuroimaging studies had found and had important demographic and clinical differences. Together, these studies demonstrate the utility of using data-driven approaches to characterize subtypes or person-specific rs-FC network characteristics and link them to specific psychiatric phenomena. However, a major gap in this emerging literature is that few studies have used this approach in SUD [3], and existing studies have not linked data-driven rs-FC subgroups or person-specific network features to clinically meaningful treatment outcomes, such as cue-induced craving or slips during treatment [12].

### Analytical plan

The current study aimed to test whether GIMME-derived subgroups and person-specific rs-FC features (within- and between-network density) were associated with three clinically-relevant outcomes in a sample of adults with TUD, (1) pre-cessation subjective cue-induced craving, and (2) daily diary-reported craving for regular cigarettes and (3) use of regular cigarettes (i.e., “slips”) during a two-week smoking cessation trial. We hypothesized that GIMME-derived group-level connectivity maps will show high connectivity within established resting-state networks [4], and connectivity between the DMN and SN, shown previously in nicotine-dependent individuals [13]. We hypothesized that subgroups of similar rs-FC, if identified, would be linked to differences in the clinical outcome measures. Although prior research has linked greater connectivity within the SN to nicotine dependence [13] and craving [14], and reduced negative connectivity between the SN and DMN with nicotine withdrawal versus satiety [15], these studies have not examined whether individual variation in connectivity is linked to clinical outcomes during treatment. Thus, in the current study, we used a data-driven approach to extend prior work to a clinical context and hypothesized that person-specific network features would be linked to clinical outcomes, such that greater positive connectivity in the SN would be linked to worse treatment outcomes (i.e., higher craving, more slips), while more negative connections between the SN and DMN would be linked to better treatment outcomes (i.e., less craving, fewer slips).

## Materials and methods

### Participants

Forty-nine participants with nicotine dependence (28 male, 21 female) were recruited as a part of a larger clinical trial of smoking cessation aids; nicotine replacement therapy and nicotine-free electronic cigarettes. This study uses data from the pre-cessation baseline MRI scan and clinical outcome measures during the two-week nicotine replacement therapy and e-cigarette smoking cessation intervention. Participants between the ages of 18 and 45 (Table 1) were recruited based on smoking nicotine cigarettes daily for at least the last six months and being willing to use nicotine replacement therapy and an e-cigarette for 2 weeks. Exclusion criteria included SUD in the last year (except cannabis, tobacco, or alcohol), current moderate or severe cannabis or alcohol use disorder, a major depressive episode in last three months, lifetime DSM-5 diagnosis of organic mental disorder, psychotic disorders, bipolar 1, positive urine drug screen (except cannabis) or alcohol breath screen at the MRI, history of head trauma with loss of consciousness >3 min, MRI contraindications, claustrophobia, and propylene glycol sensitivity/allergy. Participants provided written informed consent in accordance with the Mass General Brigham Institutional Review Board.

**Table 1:** Participant characteristics.

Measure	*N* (%)
Sex	
Male	28 (57.14)
Female	21 (42.86)
Race	
White/European American	34 (69.38)
Asian	6 (12.24)
Black/African American	5 (10.20)
More than one race	4 (8.16)
Ethnicity	
Non-Hispanic	45 (91.83)
Hispanic	4 (8.16)
**Measure**	**Mean (SD)**
Age in years	28.35 (6.36)
Fagerström test for nicotine dependence score	4.49 (1.86)
Average number of cigarettes per day	11.62 (5.34)
Age started smoking, years	17.73 (3.68)
Pack-years	6.65 (5.98)
Desire to smoke pre-MRI (1–5)	2.14 (1.06)
Desire to smoke post-MRI (1–5)	2.71 (1.16)
Desire to smoke difference	0.57 (1.32)

Pack-years refer to a numerical value of lifetime tobacco exposure, considering both intensity (packs) and duration (years), where a pack-year is twenty cigarettes used every day for one year.

### Procedure

The larger study consisted of a psychiatric interview to determine eligibility and an MRI scan prior to cessation, a check-in one week after the MRI scan, post-treatment MRI scan one week after the check-in visit, and follow-up visits 4 and 8 weeks after the post-treatment MRI. To normalize all fMRI procedures relative to the last cigarette smoked, all participants smoked one of their own cigarettes in the laboratory approximately 60 min before the MRI scan. The MRI scan included a 6-minute resting-state scan followed by a 30-minute smoking-cue reactivity task, described in detail in [16].

To assess cue-induced craving, 15 minutes before entering the scanner and immediately upon exiting the scanner, participants completed the positive and negative affect schedule (PANAS) [17], which had a single item, “desire to smoke” added to the scale. Participants rated their desire to smoke from 1 (“Very Slightly”) to 5 (“Extremely”). Cue-induced craving was measured by computing the total score post-scan minus pre-scan.

Following the MRI scan, participants were provided with nicotine patches (dose-dependent on current cigarette use) alone (*n* = 20) or nicotine patches and a nicotine-free e-cigarette (*n* = 29). Participants were instructed to refrain from smoking regular cigarettes during the two-week intervention. Participants completed daily diary assessments that assessed the number of regular cigarettes used and cigarette craving. Craving was rated from 0 (“none at all”) to 10 (“extremely high”) and was averaged to compute the mean daily cigarette craving during the treatment period. To assess the number of “slips” during the treatment, daily diary reports of the number of regular cigarettes used were summed and cross-referenced with reports obtained during the post-treatment visit to ensure accuracy.

### fMRI data collection and preprocessing

The MRI scan was conducted on a Siemens Prisma 3 T scanner with a 64-channel head coil. Resting-state fMRI was acquired using the following parameters: TR = 720 ms, TE = 30 ms, slices = 66, phase encode direction posterior to anterior, flip angle = 66°, voxel size = 2.5 × 2.5 × 2.5 mm, GeneRalized Autocalibrating Partially Parallel Acquisition (GRAPPA) factor = 2, multiband acceleration factor = 6. Multiecho multiplanar rapidly acquired gradient echo structural images were acquired with the following parameters: TR = 2500 ms, TE = 3.3, 6.98, 8.79, and 10.65 ms, flip angle = 7°, resolution = 1.33 × 1 × 1 mm. Images were preprocessed using FSL [18]. The data processing pipeline included brain extraction using BET, MCFLIRT motion correction, coregistration and normalization to MNI space, slice-time correction, spatial smoothing at 6 mm FWHM, and high-pass filtering at 0.01 Hz. Data were denoised using independent component analysis using FIX [19].

Eleven regions of interest were selected to characterize key nodes of the DMN, SN, and FPN. DMN ROIs (*n* = 2) included the medial prefrontal cortex and posterior cingulate, SN ROIs (*n* = 5) included the left and right anterior insula, dorsal anterior cingulate, and left and right nucleus accumbens, FPN ROIs (*n* = 4) included the left and right dorsolateral prefrontal cortex and left and right intraparietal sulcus. To characterize person-specific rs-FC, participant-specific ROIs were created that corresponded with established resting-state networks [20] but were scaled to individual anatomy. This was achieved by projecting a 400-area gradient-weighted Markov Random Field parcellation [20] onto individual structural scans that were processed using FreeSurfer 5.3.0., which were then coded based on spatial overlap with the Yeo et al. 17-network parcellation [21]. Parcels corresponding to the ROIs were selected and combined to make eleven subject-specific anatomical ROIs for each subject (See Supplemental Materials). Mean timeseries at each volume were extracted for each ROI and used as input for GIMME models.

### GIMME

Subgrouping GIMME (S-GIMME, version 0.7–8) was used in R to estimate individual, subgroup, and group-level maps of rs-FC. The GIMME process incorporates both contemporaneous and time-lagged (t-1) information between ROIs and autoregressive effects, allowing for the estimation of the presence of a significant connection and its directionality. The steps for S-GIMME are described previously [7, 22]. Briefly, S-GIMME begins with an empty null network and fits person-specific unified structural equation models by iteratively adding paths that contribute to better model fit, first identifying connections shared amongst the group, then subgroup, and finally individual-specific connections. GIMME is a sparse network mapping approach, meaning only paths that account for a significant amount of variance are added until excellent model fit is achieved on two out of four standard fit indices: comparative fit index (CFI ≥ 0.95), non-normed fit index (NNFI ≥ 0.95), root-mean-square error of approximation (RMSEA ≤ 0.05), and the standardized root-mean-square residual (SRMR ≤ 0.05). Additionally, paths that become non-significant with the addition of new connections are pruned.

S-GIMME begins with estimating group-level connections, which continues until there are no connections that would significantly improve a majority (75%) of individuals’ models. Following the group-level search, subgroups of individuals with similar connectivity patterns are estimated using the Walktrap community detection algorithm to compute a sparse count similarity matrix that considers the presence of the connection and its sign (positive or negative beta value). Subgroup-level connections are constructed similarly to the group-level estimation, adding paths that improve model fit for most individuals in a subgroup. Finally, GIMME estimates individual-level models by adding any additional paths.

### Cluster validation

Evaluating cluster solutions is an emerging area of research, with recent reviews highlighting the importance of evaluating cluster solutions to ensure that a given solution truly contains subjects that are more related to each other than they are to subjects outside the cluster [12]. The stability and validity of the GIMME subgrouping solution were evaluated using the R package perturbR [23]. PerturbR incrementally introduces noise to network edges while maintaining the original graph’s overall properties and compares perturbed cluster solutions with the original solution. A cluster solution is considered stable if the graph has 20% or more of its edges perturbed before the cluster solution for the rewired graph is as different as when 20% of the nodes are randomly placed into different clusters [23].

### Linking rsFC with clinical outcomes

To test whether individual-level GIMME-derived within- or between-network density (i.e., the proportion of connections within or between network nodes) was associated with clinical outcomes, separate linear regressions were conducted with the positive and negative within-network (DMN-DMN, SN-SN, FPN-FPN) and between-network (DMN-SN, SN-FPN, FPN-DMN) density as predictors of each clinical outcome. To account for variation in the total number of connections, proportions were used. Slips during treatment were modeled using negative binomial regression to account for over-dispersed count data. Craving during treatment and cue-induced craving were log-transformed to reduce heteroskedasticity. All analyses included age, sex, baseline nicotine dependence, and treatment type as covariates. Analyses were Bonferroni corrected across the within and between-network models of the three clinical outcomes of interest (i.e., *p* < 0.05/6 = 0.008).

## Results

### Model fit

All person-specific resting state networks fit the data well, according to average indexes: RMSEA = 0.0678, SRMR = 0.0395, CFI = 0.9615, and NNFI = 0.9375.

### Group-level connectivity

**Fig. 1:**
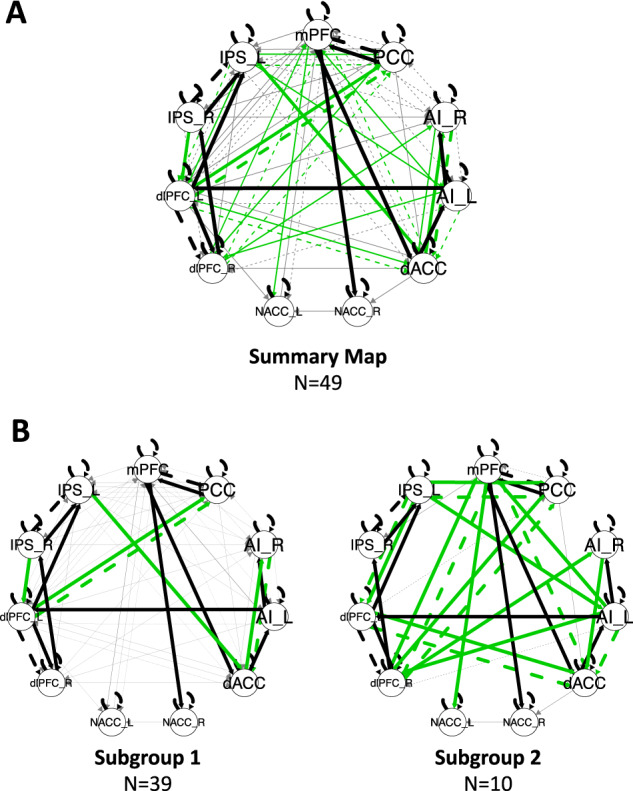
GIMME-derived resting state connectivity maps. **A** Summary connectivity map displaying significant group-level connections in black, subgroup-level connections in green, and individual-level connections in gray. Solid lines indicate contemporaneous connections, and dashed lines indicate time-lagged connections (t-1). **B** Subgroup-level connectivity maps for the two GIMME-derived subgroups.

Group-level connectivity is summarized in Fig. 1A. Contemporaneous connections were detected within the SN (*n* = 2), FPN (*n* = 4), DMN (*n* = 1), and between the SN and FPN (*n* = 1) and SN and DMN (*n* = 2). Group-level lagged connections were detected within the FPN (*n* = 2) and DMN (*n* = 1) in addition to auto-regressive effects for each ROI.

### Subgroup connectivity and validation

Two subgroups of participants were identified (Fig. 1B). The first subgroup (*n* = 39) had 6 subgroup-level connections, and the second subgroup (*n* = 10) had 18 subgroup-level connections. However, perturbR cluster validation indicated that the subgrouping solution was not stable or valid (see Supplement). Thus, we did not proceed with analyses testing whether subgroups differed on clinical outcome measures.

### Person-specific network features and clinical outcomes

We tested whether person-specific measures of within- and between-network density were associated with the three clinical outcome variables: slips and daily-diary ratings of cigarette cravings during treatment and cue-induced craving at baseline. Network metrics were only interpreted in regression models if significant connections were found for at least 25% of the sample (Table 2).

**Table 2:** Descriptive statistics of network connectivity.

	Subjects with non-zero proportions (*N*)	Mean (SD)	Range
Within-Network			
DMN-DMN pos	49	0.119 (0.019)	0.071–0.156
DMN-DMN neg	6	0.003 (0.009)	0.000–0.033
SN-SN pos	49	0.272 (0.033)	0.214–0.300
SN-SN neg	3	0.002 (0.007)	0.000–0.030
FPN-FPN pos	49	0.336 (0.039)	0.245–0.387
FPN-FPN neg	49	0.307 (0.034)	0.204–0.354
Between-Network			
DMN-FPN pos	49	0.047 (0.021)	0.029–0.102
DMN-FPN neg	48	0.043 (0.020)	0.000–0.102
DMN-SN pos	49	0.076 (0.019)	0.031–0.119
DMN-SN neg	13	0.009 (0.017)	0.000–0.059
SN-FPN pos	49	0.079 (0.026)	0.033–0.142
SN-FPN neg	16	0.011 (0.017)	0.000–0.061

To account for differences in the total number of connections, within and between-network connectivity was calculated using proportions. Not all participants had significant paths between network nodes, so we provide a count of participants with any significant paths within or between-network paths, in addition to the mean, standard deviation, and range of the network density metrics. Network metrics were only interpreted in regression models if there were significant connections for at least 25% of the sample.

**Fig. 2:**
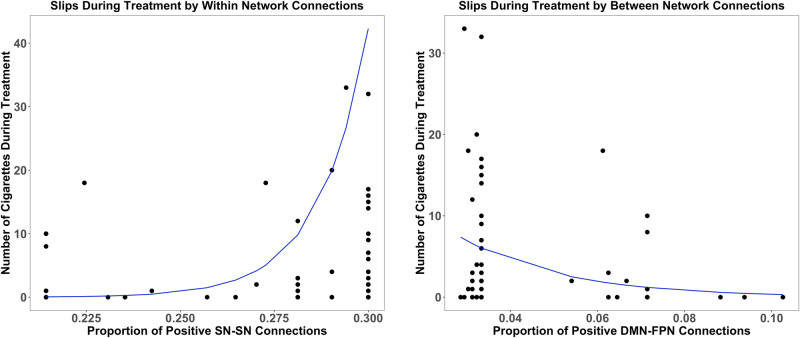
Associations between within- and between-network connectivity and slips during treatment. Positive SN-SN density was associated with more slips, while positive DMN-FPN density was associated with fewer slips.

Slips during treatment was positively associated with the density of positive SN-SN connections (χ^2^ = 21.841, *p* < 0.001; b = 69.796, s.e. = 23.100, *p* < 0.003). The density of positive DMN-FPN connections was associated with fewer slips during treatment (χ^2^ = 26.195, *p* < 0.001; b = −42.318, s.e. = 14.565, *p* < 0.004) (Fig. 2). These effects were unchanged when adding baseline cigarette use as a covariate in the model.

**Fig. 3:**
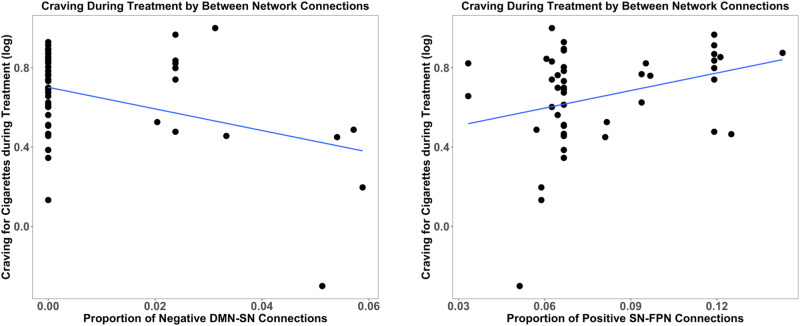
Associations between between-network connectivity and craving during treatment. Negative DMN-SN density was associated with lower craving during cessation, while positive SN-FPN density was associated with higher craving during cessation.

Craving for regular cigarettes during treatment was associated with between-network connectivity (R^2^ = 0.447, F(10,37) = 2.990, *p* < 0.007). The density of negative DMN-SN connections was related to decreased craving during treatment (β = −0.480, *p* < 0.002) (Fig. 3) and the density of positive SN-FPN connections (β = 0.528, *p* < 0.005) was related to increased craving during treatment. The density of negative SN-FPN connections was associated with increased craving during treatment (β = 0.453, *p* < 0.010) and more cue-induced craving (β = 0.359, *p* = 0.043), but these effects did not survive multiple comparisons correction.

## Discussion

The study used a novel data-driven approach to characterize group-, subgroup-, and person-specific rs-FC features in adults with nicotine dependence and linked these features to clinical measures: cue-induced craving and craving and slips during treatment. As hypothesized, we found significant within-network connectivity in the SN, DMN, and FPN for all subjects and significant between-network connections in the SN-DMN and FPN-SN. However, the novel contribution of the current study is linking person-specific rs-FC and clinical outcomes.

Individuals with a greater density of positive SN-SN connections (i.e., more connectivity between SN regions) experienced more slips (i.e., smoked more cigarettes) during treatment. These findings were driven by dorsal anterior cingulate-insula connectivity since few participants had significant connectivity between the nucleus accumbens and other SN ROIs. Consistent with this finding, several studies have shown that greater connectivity within the SN, particularly the anterior insula and dorsal anterior cingulate, is linked to nicotine dependence [13], more frequent cigarette use [24], greater smoking cue-reactivity [14, 25], and predicts relapse [26–28]. Our findings, when combined with existing literature, suggest that individuals with greater SN connectivity represent a particularly vulnerable group, as they may experience greater sensitivity to smoking cues in the environment and may be at increased risk for relapse during quit attempts.

Conversely, individuals with a greater density of positive DMN-FPN connections experienced fewer slips during treatment. Relatively little prior work has reported significant connectivity between these networks in samples with SUD. However, regions of the FPN coactivate with the DMN during tasks requiring regulation of internally oriented processes [29] and have been suggested to play a role in regulating introspective processes [30]. Thus, greater DMN-FPN connectivity may help individuals better guide behavior in the context of heightened internally oriented processing (e.g., craving, withdrawal) during quit attempts. Additional research is needed to understand whether there is a robust link between DMN-FPN connectivity and nicotine dependence.

Consistent with hypotheses, individuals with a greater density of negative SN-DMN connections (i.e., more anticorrelated connectivity) reported lower craving for regular cigarettes during treatment. Prior work has shown that reduced anticorrelated activity between the SN-DMN after a 24-hour abstinence period was associated with higher levels of craving [15]. Our findings are consistent with and extend prior research to link SN-DMN connectivity to craving during a two-week smoking cessation treatment. Additionally, participants in our study received pharmacologic intervention (i.e., NRT scaled to baseline nicotine use), whereas participants in Lerman et al (2014) were in an acute nicotine withdrawal state. Thus, the consistency of our findings may suggest that links between SN-DMN connectivity and craving are related to non-pharmacological aspects of abstinence.

A growing body of research suggests that enhanced connectivity between the SN and DMN is associated with more focus on internal affective states. For example, baseline craving was associated with enhanced connectivity ventral AI and DMN [14], and regions of the SN may enhance the self-referential processing of smoking cues by modulating the DMN [31]. Moreover, the connectivity between regions of the SN and DMN is enhanced during abstinence [32], and conversely, nicotine administration acutely reduces the time spent in the frontoinsular DMN [33]. Together, these findings highlight that SN-DMN interactions are modulated by internally oriented affective states such as craving. While other findings demonstrate a pharmacological effect of nicotine (or nicotine abstinence) impacts SN-DMN connectivity [15, 32, 33], a novel contribution of the current work is that variance in SN-DMN connectivity can be detected even following recent smoking and is predictive of treatment outcomes. Combined with prior findings suggesting that enhanced DMN-SN connectivity may be linked with greater craving, our findings suggest that greater anticorrelated connectivity between the SN and DMN may be protective for individuals attempting to reduce smoking.

An unexpected finding was that the density of positive SN-FPN connections was related to more craving during treatment. Regions of the SN, especially the dorsal AI, show reactivity to external smoking cues [14] and functional coupling with goal-directed networks, including the FPN [34]. The SN has also been proposed to be a dynamic switch between the DMN and FPN, modulating internally and externally-oriented attention [34, 35]. Individuals with more positive SN-FPN connectivity may be more likely to engage with external cues in the environment, leading to increased craving during quit attempts. More negative SN-FPN connectivity was also related to increased craving, but this effect did not survive multiple comparisons correction. Recently, Drossel et al. found that distinct subtypes of individuals with SUDs were linked to different patterns of rs-FC. In “reward-type” individuals (i.e., higher approach-related behavior), substance use was related to decreased connectivity in the FPN and SN, while in “cognitive-type” (i.e., lower executive function), substance use was related to increased connectivity in the FPN and SN [36]. This suggests that different individuals have distinct neurobiological pathways contributing to their craving. Further research will advance intervention approaches for important clinical targets (i.e., craving) but are tailored to individual differences in neurobiological pathways.

### Strengths and limitations

The current study’s strengths include using a novel technique that merges nomothetic and idiographic modeling approaches to link group- subgroup- and person-specific rs-FC features with clinical treatment outcomes in individuals with TUD. It is one of the first to use this approach in a SUD sample while also assessing the robustness and validity of the data-driven subgroups. However, it also has several limitations. First, the six-minute resting-state scan was relatively brief, however, with a TR = 720, this seemingly short sequence allowed us to contribute 500 data points to the GIMME analysis, well above the time points tested in a prior validation study [37], which demonstrate robust S-GIMME results with time points as low as 60. Indeed, signal-to-noise ratios could be impacted by factors such as the multi-band acceleration factor [38] and smaller voxel size [39] that our current study used, although this is somewhat balanced by the fact that the TR is sufficiently short that respiration is unaliased in the data, and can be fully removed. However, further replication and validation of our findings is desirable in datasets with longer scan durations. Second, although we identified two subgroups, one with relatively more between-network connections than the other, these subgroups were not robust or stable based on established subgroup validation approaches [23]. A recent review [12] highlights that most existing studies that identify brain-based subtypes of psychopathology do not test for the validity of the subtyping solution and do not assess the clinical utility of the approach by linking them with clinical treatment outcomes. Although the sample size of the current study (*n* = 49) is larger than the minimum recommended sample for S-GIMME (n≥25), an important next step in this work would be external validation to determine whether a similar cluster solution of rs-FC is present in larger clinical trials of adults with TUD. Additionally, a larger sample size may have improved the power to detect associations between GIMME-derived network features and clinical outcomes. This point is especially relevant for the finding linking anticorrelated DMN-SN connectivity with craving, as only thirteen subjects had significant anticorrelated SN-DMN activity. Nevertheless, we are encouraged by our results being consistent with prior studies [15] and theory [40]. Finally, the smoking cessation trial was brief, and it is unclear whether associations between baseline rs-FC would predict longer-term treatment outcomes.

## Conclusion

This study used a novel data-driven approach to model within and between-network rs-FC in adults with nicotine dependence and linked these features to clinical outcomes during a two-week NRT-assisted quit attempt. The GIMME approach allowed us to parse neurobiological heterogeneity by identifying patterns of rs-FC that were shared across the sample while also modeling significant connections that are unique to only a subset of participants or an individual participant and linking these rs-FC patterns to clinical outcomes. We found that individuals with more SN connectivity had more slips during treatment while individuals with more FPN-DMN connectivity had fewer slips. Greater density of anticorrelated DMN-SN connections was also linked to lower cigarette craving during treatment, while positive SN-FPN connectivity was linked to greater craving. These results extend prior research on the importance of SN, DMN, and FPN network connectivity in nicotine dependence by linking individual variation in intra and internetwork connectivity to treatment outcomes and suggest that high SN connectivity may be a risk factor for smoking relapse, while greater positive DMN-FPN and anticorrelated SN-DMN connectivity are protective factors during nicotine-cessation treatment.

### Supplementary information

Supplement
