# Supplementary material for: Data-driven connectivity profiles relate to smoking cessation outcomes
Source: Neuropsychopharmacology. 2024 Jan 27;49(6):1007–13. doi: 10.1038/s41386-024-01802-9 (PMC11039768; doi:10.1038/s41386-024-01802-9)
Supplement: Supplementary file 1 — Supplement [file 41386_2024_1802_MOESM1_ESM.docx]

**Supplemental Materials**

**Data-Driven Connectivity Profiles Relate to Smoking Cessation Outcomes**

Laura Murray, Blaise B. Frederick, Amy C. Janes

**Supplemental Table 1: Regions of Interest for GIMME Analyses**

| **Region of Interest** | **Schaefer Look Up Table Value** | **Image** |
| --- | --- | --- |
| ***Salience Network*** | | |
| Left Anterior Insula | 1104, 1105, 1106 | 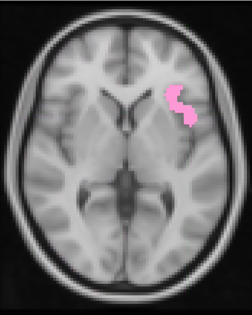 |
| Right Anterior Insula | 2109, 2110 | 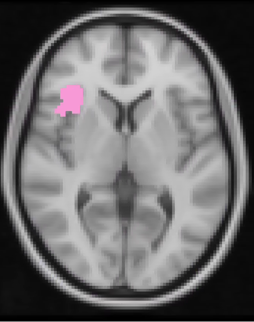 |
| dACC | 2112, 2111, 1108 | 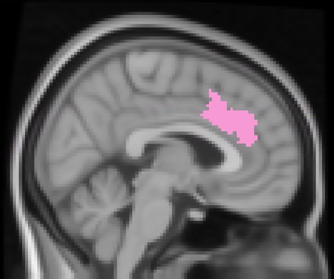 |
| ***Default Mode Network*** | | |
| mPFC | 2168, 2170, 2171, 1161, 1162, 1163, 1164 | 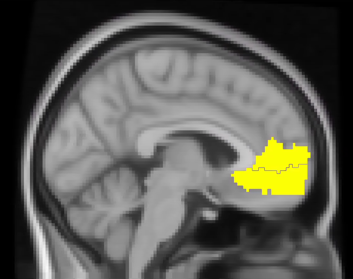 |
| PCC | 2163, 2164, 2165, 2167, 1154, 1155, 1156, 1157, 1159, 1160 | 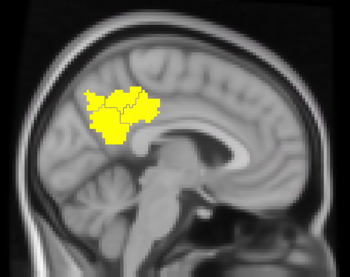 |
| ***Frontoparietal Network*** | | |
| Left IPS | 1122, 1123, 1124, 1126 | 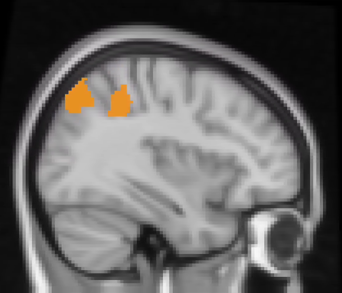 |
| Right IPS | 2125, 2126, 2127, 2128 | 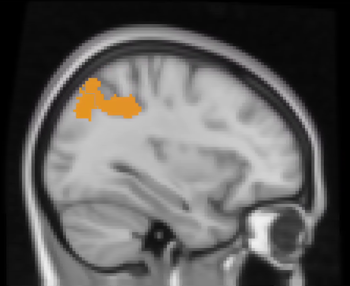 |
| Left dlPFC | 1128, 1129, 1130, 1131, 1132 | 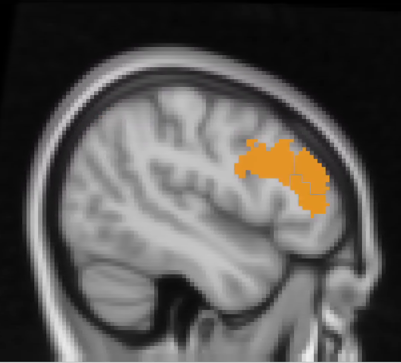 |
| Right dlPFC | 2130, 2131, 2132, 2133 | 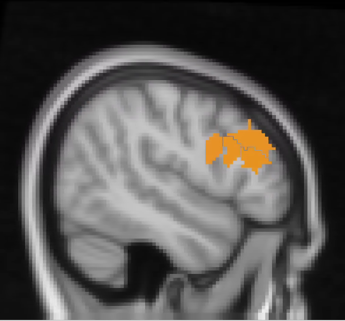 |

**Supplemental Table 1: Regions of Interest for GIMME Analyses.** Regions of Interest (ROIs) were selected from the Schaefer 400 region parcellation of the Yeo 2011 17-Network atlas (Schaefer et al., 2018). Parcels numbers from the Schaefer look up table (available at https://github.com/ThomasYeoLab/CBIG/blob/master/stable_projects/brain_parcellation/Schaefer2018_LocalGlobal/Parcellations/project_to_individual/Schaefer2018_400Parcels_17Networks_order_LUT.txt) are indicated in column 2 were combined to create each ROI. ROIs were scaled to individual anatomy using freesurfer according to guidelines in Schaefer et al 2018, however for visual purposes the ROIs are displayed on the MNI152 2mm template.

**Subgroup Connectivity**

Two subgroups of participants were identified. The first subgroup (n = 39) had 6 subgroup-level connections, including contemporaneous within-network connections in the SN (dACC to right AI) and FPN (right IPS to left dlPFC) and between-network connections in the DMN and FPN (left dlPFC to PCC) and SN and FPN (left IPS to dACC). Subgroup-level lagged connections included a within-network SN connection (right AI to dACC) and an FPN-DMN connection (left dlPFC to PCC).

The second subgroup (n=10) had 18 subgroup-level connections, including one contemporaneous within-network connection in the SN (right AI to dACC) and ten contemporaneous between-network connections, in the DMN and FPN (right dlPFC to mPFC, right dlPFC to PCC, left IPS to PCC), the SN and FPN (left AI to right dlPFC, left dlPFC to dACC, right dlPFC to right AI, left IPS to left AI, left IPS to left dlPFC), and the SN and DMN (left AI to mPFC, mPFC to left NACC). Subgroup-level lagged connections included within-network connections in the SN (left AI to dACC) and FPN (left IPS to left dlPFC), and between-network connections between the DMN and FPN (right dlPFC to mPFC, right dlPFC to PCC, left IPS to PCC), SN and FPN (left dlPFC to dACC), and SN and DMN (dACC to mPFC).

**Cluster Validation**

The validity of the S-GIMME subgroups was assessed using the perturbR package in R. (Gates et al., 2019). Stability is assessed with the Hubert-Arabie Adjusted Rand Index (ARI) and Variation of Information (VI) metrics, which describe the degree to which two community solutions differ. An ARI of at least 0.80 is generally considered evidence of similarity in cluster solutions, and a VI of at least 0.20 is considered evidence of cluster stability. A third metric, modularity, is assessed using perturbR via Monte Carlo simulations that provide a distribution of modularity values from cluster solutions using random matrices with similar overall weight as the original. The cluster solution is considered valid if modularity for the original solution is greater than or equal to the 95th percentile of modularity obtained from random graphs (Gates et al., 2019).

According to VI, ARI, and modularity, the cluster solution attained was not stable nor valid (Supplementary Fig 1). Based on VI, 17% of edges had to be perturbed before 20% of participants were placed into different clusters than the original solution. Cluster solutions are considered robust if the matrix had 20% of its edges perturbed (α ≥ 0.20) before intersecting with the line representing 20% of the participants being in different clusters. The ARI was 0.311 indicating low similarity of original cluster solution and the solution with 20% of the edges perturbed. Modularity attained (0.034) was not better than expected by chance (95th percentile of random graphs = 0.092), suggesting that clusters are not well defined and participants in different clusters may have more in common than expected if clusters were truly distinct. Thus, based on VI, ARI, modularity indices, the cluster solution is not robust and thus we did not investigate subgroup differences in clinical outcomes.

Supplementary Figure 1. PerturbR Output. A). Variation of Information (VI) plot. The x-axis is the proportion of perturbed edges. Horizontal Line represents point at which 20% of nodes are randomly swapped between clusters. Black dots represent perturbed paths based on original subgroup solution. Red dots represent the perturbed solution. The figure indicates that, based on VI, 17% of edges had to be perturbed before 20% of participants were placed into different clusters than the original solution. Cluster solutions are considered robust if the matrix had 20% of its edges perturbed (α ≥ 0.20) before intersecting with the line representing 20% of the participants being in different clusters. (B) The Adjusted Rand Index (ARI) was 0.311 indicating low similarity of original cluster solution and the solution with 20% of the edges perturbed. (C) Modularity attained (0.034) was not better than expected by chance (95th percentile of random graphs = 0.092), suggesting that clusters are not well defined and participants in different clusters may have more in common than expected if clusters were truly distinct. Thus, based on VI, ARI, modularity indices, the cluster solution is not robust and thus we did not investigate subgroup differences in clinical outcomes. The perturbR method is discussed in detail in Gates et al., 2019, and a tutorial is available at https://cran.r-project.org/web/packages/perturbR/vignettes/perturbR-vignette.html.

**References**

Gates, K.M., Fisher, Z.F., Arizmendi, C., Henry, T.R., Duffy, K.A., Mucha, P.J., 2019. Assessing the robustness of cluster solutions obtained from sparse count matrices. Psychological methods 24(6), 675.

Schaefer, A., Kong, R., Gordon, E.M., Laumann, T.O., Zuo, X.-N., Holmes, A.J., Eickhoff, S.B., Yeo, B.T., 2018. Local-global parcellation of the human cerebral cortex from intrinsic functional connectivity MRI. Cerebral cortex 28(9), 3095-3114.
